# Risk factors for multiple bronchoalveolar lavage procedures in children with severe or refractory *Mycoplasma pneumoniae* pneumonia

**DOI:** 10.1016/j.jped.2026.101557

**Published:** 2026-05-22

**Authors:** Chaolei Yang, Guitao Li, Ying Zhu, Yong Wang, Lei Zhang

**Affiliations:** a Fuyang Normal University, Fuyang Medical College, Fuyang, Anhui, PR China; b The Affiliated Fuyang People's Hospital of Anhui Medical University, Department of Pediatrics, Fuyang, Anhui, PR China; c The Second Affiliated Hospital of Fuyang Normal University, Department of Respiratory Medicine, Fuyang, Anhui, PR China

**Keywords:** Children, *Mycoplasma pneumoniae* pneumonia, Bronchoscopy, Bronchoalveolar lavage

## Abstract

**Objective:**

To identify factors influencing how often bronchoalveolar lavage (BAL) is performed in children with severe or refractory *Mycoplasma pneumoniae* pneumonia (MPP) and provide evidence to guide early intervention.

**Methods:**

Clinical data for 271 children with severe or refractory MPP who underwent BAL at the studied institution between May 1, 2023, and April 30, 2024, were retrospectively divided according to the number of BAL procedures into a single-treatment group (STG, n = 219) and a multiple-treatment group (≥ 2 times; MTG, n = 52). Risk factors for multiple BAL procedures in these children were identified by logistic regression and receiver-operating characteristic curve analyses.

**Results:**

Peak body temperature was significantly higher, duration of fever was significantly longer, and pulmonary consolidation, pleural effusion, atelectasis, mucus plug formation, and pulmonary lesions involving two or more lung lobes were significantly more common in the MTG than in the STG (all *P* < 0.05). The white blood cell count, neutrophil percentage, and C-reactive protein (CRP), lactate dehydrogenase (LDH), fibrinogen, and D-dimer levels were significantly higher and the lymphocyte percentage was significantly lower in the MTG (*P* < 0.05). Logistic regression and receiver-operating characteristic curve analyses showed that mucus plug formation, CRP ≥ 25.68 mg/L, LDH ≥ 374.90 U/L, and D-dimer ≥ 1.22 mg/L were independent risk factors for multiple BAL in children with severe or refractory MPP.

**Conclusion:**

Mucus plug formation and increased CRP, LDH, and D-dimer levels are important risk factors for multiple BAL in children with severe or refractory MPP. Pediatricians should strengthen management and early intervention in these children to avoid the harm caused by multiple BAL procedures.

## Introduction

*Mycoplasma pneumoniae* pneumonia (MPP) is one of the most common types of community-acquired pneumonia in children aged 5 years or older in China, accounting for more than 40% of pediatric cases of community-acquired pneumonia in epidemic years [1]. Most cases of *M. pneumoniae* in China in recent years have involved macrolide-resistant strains, leading to an increase in the proportion of children with severe or refractory MPP [[2], [3], [4]]. The enzyme lactate dehydrogenase (LDH) is present in various cells throughout the body and catalyzes the reversible conversion of lactate to pyruvate. It has been suggested that the LDH level is an important indicator of the need for administration of glucocorticoids in children with MPP [5]. C-reactive protein (CRP) is widely used to evaluate inflammatory status and the response to infection. Its elevation is particularly significant in *M. pneumoniae* infection, especially in cases with large parenchymal lesions in the lungs and pleural effusion [6]. D-dimer is a specific fibrinolytic marker that is used to monitor inflammation and the severity of infection. Its elevation indicates a hypercoagulable state. Accumulating evidence indicates that coagulation status is closely associated with the inflammatory response, which is of significance in children with MPP [7]. Bronchoalveolar lavage (BAL) has become a major aid to successful treatment in children with severe or refractory MPP. It is a therapeutic intervention that can clear endogenous foreign bodies in the lower respiratory tract, including airway secretions, mucus plugs, and blood clots, and improve ventilation, thereby facilitating the resolution of local inflammatory lesions. Some children require multiple BAL procedures to effect a clinical cure [8,9]. However, as an interventional procedure, BAL is associated with risks and complications, including hypoxia, hemorrhage, bronchospasm, arrhythmia, and pneumothorax [[10], [11], [12], [13]]. How to reduce the need for multiple BAL procedures in children with severe or refractory MPP has become a key concern.

In this study, the authors retrospectively analyzed the clinical data for a large group of children with severe or refractory MPP to identify risk factors for multiple BALs. The aims of the study were to facilitate early identification and management of these patients, mitigate the adverse effects of repeated BAL procedures, and alleviate the disease-related treatment burden and distress.

## Methods

### Study participants

**Figure 1:**
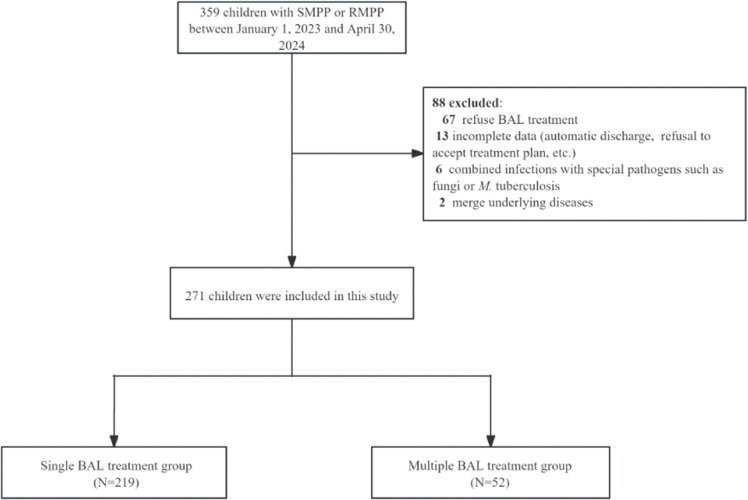
Flow diagram showing the process used to select the study participants. BAL, bronchoalveolar lavage; RMMP, refractory *Mycoplasma pneumoniae* pneumonia; SMMP, severe *Mycoplasma pneumoniae* pneumonia.

Children with severe or refractory MPP who underwent BAL in the Department of Pediatrics, Fuyang People's Hospital between May 1, 2023, and April 30, 2024, were retrospectively identified. The children were divided according to the number of BAL procedures performed during the course of their disease into a single-treatment group (STG, n = 219) and a multiple-treatment group (≥ 2 times; MTG, n = 52) (Figure 1). Informed consent was obtained from each child’s guardian before performing fiberoptic bronchoscopy.

### Inclusion criteria

(1) Age 28 days to 16 years.
(2) Diagnostic criteria for severe or refractory MPP met according to the 2023 guidelines for the diagnosis and treatment of MPP in children (https://www.gov.cn/zhengce/zhengceku/2023-02/16/content_5741770.htm). Severe MPP is defined as follows: persistent high fever (39°C) for ≥ 5 days or fever lasting ≥ 7 days with no downward trend in peak temperature; presence of one or more of wheezing and tachypnea, dyspnea, chest pain, and hemoptysis; extrapulmonary complications not meeting the criteria for critical illness; resting fingertip SpO_2_ of ≤ 0.93 on room air; imaging demonstrating involvement of two-thirds or more of a single lung lobe with homogeneous high-density consolidation or high-density consolidation in two or more lung lobes (regardless of size of the affected area); moderate to large pleural effusion or localized manifestations of bronchiolitis (diffuse bronchiolitis observed in one or both lungs or ≥ 4/5 lobes, potentially complicated by bronchitis and formation of mucus plugs leading to atelectasis); progressively worsening clinical symptoms, with imaging demonstrating an increase in the extent of lesions by more than 50% within 24–48 hours; and a significant elevation in one of CRP, LDH, or D-dimer. Refractory MPP in pediatric patients is defined as persistent fever, progressive deterioration of clinical signs and pulmonary imaging findings, or development of extrapulmonary complications despite receiving standard macrolide antibiotic treatment for ≥ 7 days.
(3) Detection of *M. pneumoniae* as the main pathogen in BAL fluid by multiple targeted next-generation sequencing technology.

### Exclusion criteria

(1) An underlying condition, such as immunodeficiency, hematological disease, cardiovascular disease, or renal disease.
(2) Incomplete data.
(3) Refusal to undergo BAL.
(4) Fungal infection or pathogens such as *Mycobacterium tuberculosis*.

### Data collection

Demographics (including age, sex, and season of onset) and clinical data (including duration of fever and length of hospital stay) were collected from the electronic medical records. Laboratory data obtained on the first day of admission were also collected, including the white blood cell count, neutrophil percentage, myocardial enzymes, liver function, coagulation status, and CRP, LDH, and D-dimer levels. Imaging data, including for pleural effusion, atelectasis, pulmonary consolidation, and inflammation involving the lung lobes, were also retrieved, as were the results of bronchoscopy, including the presence or absence of mucus plugs.

### Bronchoalveolar lavage and detection of pathogens

**Figure 2:**
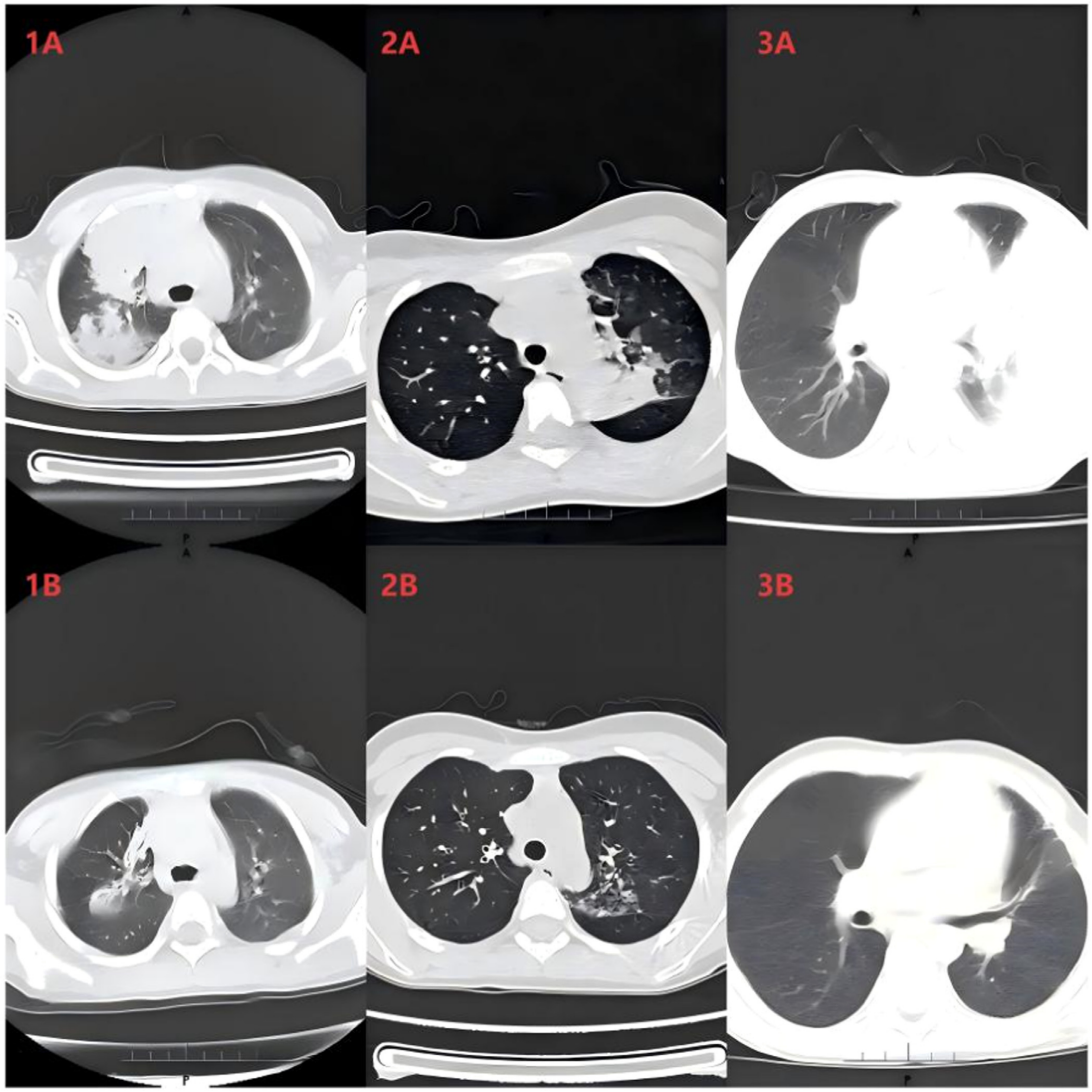
Findings on chest computed tomography before and after BAL. (**1A**) Case 1 (pre-BAL). (**1B**) Case 1 (2 weeks post-BAL). (**2A**) Case 2 (pre-BAL). (**2B**) Case 2 (1 week post-BAL). (**3A**) Case 3 (pre-BAL). (**3B**) Case 3 (2 weeks post-BAL). BAL, bronchoalveolar lavage

All patients scheduled for BAL at the studied institution receive conventional anti-infective and anti-inflammatory therapy after admission. BAL via bronchoscopy is recommended for children presenting with evidence of atelectasis or impaired ventilation on chest radiographs or pulmonary computed tomography scans and those meeting the diagnostic criteria for refractory or severe MPP. If the patient still has a fever with no improvement of clinical symptoms or progressive worsening of pulmonary imaging findings 5 days after the initial BAL, a second BAL is performed. The patient is then evaluated by two pediatricians with qualifications of attending physician or above to reduce the risk of subjective judgment based on symptoms. In principle, BAL is performed no more than three times during the entire course of treatment. Children fast for 6–8 hours before the procedure, and routine pre-procedure examinations are performed to rule out any contraindications. The most severely affected lung segment or lobe is identified based on findings from chest radiography or computed tomography to guide targeted lavage. A bronchoscope (Pentax, Tokyo, Japan) with an external diameter of 2.8 mm, 3.6 mm, or 4.9 mm is selected according to the patient’s age and body weight. A combined anesthesia regimen of “progressive local anesthesia during bronchoscope insertion” (topical local anesthesia) and conscious sedation is used during the procedure. Patients receive 2% lidocaine via a nebulizer before the insertion of the bronchoscope. After the bronchoscope is inserted, 1–2 mL of 2% lidocaine is sprayed sequentially on the larynx, glottis, trachea, and left and right main bronchi; local administration can be repeated if necessary, with the total dose of lidocaine not exceeding 7 mg/kg. Midazolam (0.1–0.3 mg/kg, maximum dose 10 mg) is administered intravenously for sedation. Spontaneous breathing is maintained throughout the procedure. The bronchoscope is inserted through one nostril, and oxygen supplementation is provided via a nasal catheter in the other nostril. The total lavage volume is determined according to the patient’s body weight. For children weighing <20 kg, the total volume is 3 mL/kg, divided into three equal aliquots for instillation; for children weighing ≥ 20 kg, each aliquot is 20 mL, with the maximum total lavage volume not exceeding 3 mL/kg. After negative pressure suction, 5–10 mL of BAL fluid is collected in a sterile container and transported at 2–8°C to the laboratory for testing. The primary purpose of BAL is to clear the airways, whereas pathogen detection assists clinicians with the identification of causative pathogens. After BAL treatment, significant improvement in pulmonary lesions was observed in the pediatric patient (Figure 2). All BAL procedures described above comply with the requirements of the Clinical Practice Guideline for Bronchoalveolar Lavage in Chinese Children (2024) [14].

### Statistical analysis

Continuous data conforming to a normal distribution are expressed as the mean ± standard deviation and were compared between the study groups using the *t*-test. Continuous data that were not normally distributed are shown as the median and interquartile range [IQR] and were compared between groups using the Mann–Whitney *U* test. Numerical data are expressed as the rate (percentage) and were compared between groups using the chi-squared test or Fisher’s exact test. Potential risk factors for multiple BAL procedures identified to be statistically significant in univariate analysis (*P* < 0.05) were entered into a multivariate logistic regression model. The prediction performance of each risk factor identified by multivariate logistic regression was assessed using receiver-operating characteristic (ROC) curve analysis. The ROC curves were plotted using GraphPad (GraphPad Software Inc., San Diego, CA, USA), and the cut-off values for maximum sensitivity and specificity were computed using Youden’s method. All statistical analyses were performed using SPSS 25.0 (IBM Corp., Armonk, NY, USA). A *P*-value of < 0.05 was considered statistically significant.

## Results

### Clinical data

The study included 271 children, comprising 145 boys and 126 girls (male-to-female ratio, 1.11:1). There were 219 patients in the STG (114 boys, 105 girls; average age 6.55 ± 2.71 years) and 52 in the MTG (31 boys, 21 girls; average age 7.02 ± 2.06 years). There was no statistically significant difference in sex, age, season of onset, duration of disease before the first bronchoscopic intervention, or *M. pneumoniae* DNA load between the two study groups (all *P* > 0.05). However, there were significant between-group differences in peak body temperature, duration of fever, pulmonary consolidation, complications of pleural effusion, atelectasis, and mucus plug formation, and pulmonary lesions involving two or more lung lobes (*P* < 0.05) (Table 1).

**Table 1:** Comparison of demographic and clinical data according to number of bronchoalveolar lavage procedures performed.

**Variable**	**STG (n = 219)**	**MTG (n = 52)**	**χ^2^/t/Z**	***P***
Gender [n (%)]			0.966	0.326
Male	114 (52.1)	31 (59.6)		
Female	105 (47.9)	21 (40.4)		
Onset season [n (%)]			4.270	0.234
Spring	14 (6.4)	3 (5.8)		
Summer	51 (23.3)	6 (11.5)		
Autumn	85 (38.8)	21 (40.4)		
Winter	69 (31.5)	22 (42.3)		
Age (years)	6.55±2.71	7.02±2.06	−1.382	0.170
Fever peak [M (P_25_, P_75_), °C]	39.2 (38.9, 39.7)	39.7 (39.0, 40.0)	−3.704	<0.001
Duration of fever (days)	6.16±3.73	10.17±4.42	−6.717	<0.001
Duration of disease before the first bronchoscopy intervention (days)	9.79±4.41	9.52±3.94	0.399	0.690
Pulmonary consolidation [n (%)]	80 (36.5)	36 (69.2)	18.355	<0.001
Complicated with pleural effusion [n (%)]	19 (8.7)	27 (51.9)	55.769	<0.001
Atelectasis [n (%)]	9 (4.1)	18 (34.6)	43.596	<0.001
Mucus plug [n (%)]	41 (18.7)	43 (82.7)	80.401	<0.001
Pulmonary lesions involving ≥ 2 lung lobes [n (%)]	85 (38.8)	36 (69.2)	15.733	<0.001
*M. pneumoniae* DNA load	52934.23±30364.41	52711.13±10966.02	0.052	0.958

MP, *Mycoplasma pneumoniae*; MTG, multiple-treatment group; STG, single-treatment group.

### Laboratory data

The white blood cell count, neutrophil percentage, and CRP, LDH, fibrinogen, and D-dimer levels were significantly higher, and the lymphocyte percentage was significantly lower in the MTG than in the STG (all *P* < 0.05). There was no significant between-group difference in hemoglobin, platelet count, alanine aminotransferase, aspartate aminotransferase, creatine kinase, creatine kinase isoenzyme, prothrombin time, or activated partial thromboplastin time (all *P* > 0.05) (Table 2).

**Table 2:** Comparison of laboratory data according to number of bronchoalveolar lavage procedures performed.

**Variables**	**STG (n=219)**	**MTG (n=52)**	**t/Z**	***P***
WBC (×10^9^/L)	8.19±2.95	9.46±3.99	−2.151	0.035
N%	62.98±12.26	69.91±11.78	−3.689	<0.001
L%	28.48±10.90	22.98±10.91	3.271	0.001
HGB (g/L)	122.92±15.51	122.90±11.23	0.007	0.994
PLT (×10^12^/L)	285.10±91.23	265.04±84.03	1.446	0.149
CRP (mg/L)	14.01±12.91	44.94±36.26	−6.059	<0.001
ALT (U/L)	16.63±9.89	19.29±9.34	−1.760	0.080
AST (U/L)	31.28±12.64	34.88±11.54	−1.876	0.062
CK (U/L)	87.67±66.52	95.89±75.25	−0.781	0.436
CK-MB ( U/L)	2.34 (1.54, 3.33)	2.18 (1.67, 3.44)	0.271	0.787
LDH (U/L)	321.26±88.24	491.07±146.69	−8.009	<0.001
PT (s)	12.01±0.87	12.28±0.89	−1.946	0.053
APTT (s)	30.90±8.86	30.32±6.25	0.450	0.653
FIB (g/L)	3.37 (2.83, 4.00)	3.66 (3.10, 4.61)	−2.303	0.021
D-D (mg/L)	0.56 (0.32, 1.09)	3.11 (1.75, 7.33)	−8.636	<0.001

ALT, alanine aminotransferase; APTT, activated partial thromboplastin time; AST, aspartate aminotransferase; CK, creatine kinase; CK-MB, creatine kinase isoenzyme; CRP, C-reactive protein; D-D, D-dimer; FIB, fibrinogen; HGB, hemoglobin; LDH, lactate dehydrogenase; L%, lymphocyte percentage; MTG, multiple treatment group; N%, neutrophil percentage; PLT, platelet count; PT, prothrombin time; STG, single-treatment group; WBC, white blood cell count.

### Multivariate logistic regression analysis

Multivariate logistic regression analysis revealed that mucus plug formation and elevated CRP, LDH, and D-dimer levels were strong risk factors for multiple BAL procedures in children with severe or refractory MPP (Table 3).

**Table 3:** Risk factors for multiple bronchoalveolar lavage procedures identified by multivariate logistic regression analysis.

Variables	*B*	*SE*	*Wald*	*P*	Odds ratio 95% confidence interval (CI)
Mucus plug	2.622	0.600	19.073	<0.001	13.756 (4.242∼44.612)
CRP	0.061	0.016	15.097	<0.001	1.063 (1.031∼1.096)
LDH	0.006	0.002	6.605	<0.001	1.006 (1.001∼1.010)
D-D	0.306	0.082	14.097	<0.001	1.358 (1.158∼1.594)

CI, confidence interval; CRP, C-reactive protein; D-D, D-dimer; LDH, lactate dehydrogenase; OR, odds ratio; SE, standard error.

### ROC curve analysis

**Figure 3:**
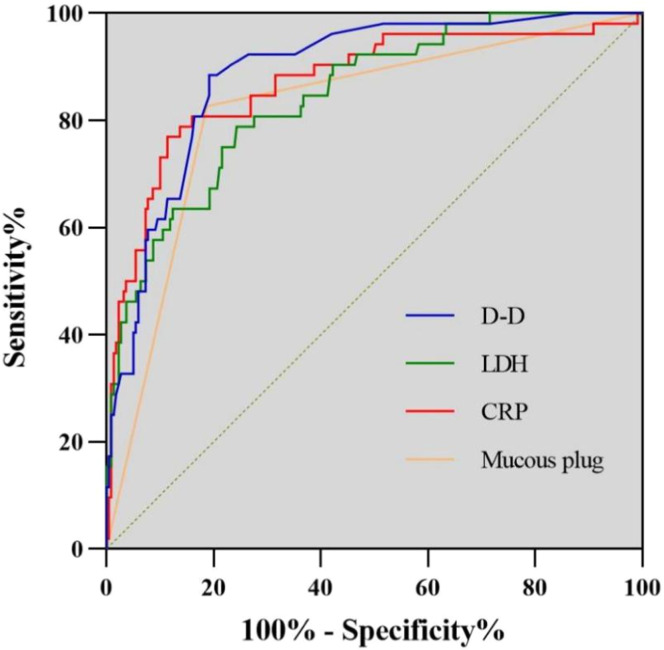
Receiver-operating characteristic curves for mucus plug formation and CRP, LDH, and D–D levels as predictors of multiple bronchoalveolar lavage procedures. CRP, C-reactive protein; D–D, D-dimer; LDH, lactate dehydrogenase.

ROC curve analysis confirmed that mucus plug formation and CRP, LDH, and D-dimer levels had good efficacy for predicting the need for multiple BAL procedures. The cut-off values were ≥ 25.68 mg/L for CRP, ≥ 374.90 U/L for LDH, and ≥ 1.22 mg/L for D-dimer; the corresponding areas under the curve were 0.819, 0.871, 0.845, and 0.885 with respective values of 0.827, 0.769, 0.788, and 0.904 for sensitivity and 0.812, 0.885, 0.757, and 0.794 for specificity (Figure 3, Table 4).

**Table 4:** Results of receiver-operating characteristic curve analysis.

Variables	Cut-off	AUC	Sensitivity	Specificity	*P*	95% CI
Mucus plug	—	0.819	0.827	0.812	<0.001	0.753∼0.886
CRP	25.68 mg/L	0.871	0.769	0.885	<0.001	0.810∼0.932
LDH	374.90 U/L	0.845	0.788	0.757	<0.001	0.787∼0.902
D-D	1.22 mg/L	0.885	0.904	0.794	<0.001	0.837∼0.933

AUC, area under the curve; CI, confidence interval; CRP, C-reactive protein; D-D, D-dimer; LDH, lactate dehydrogenase.

## Discussion

Almost all children with MPP who require BAL present with severe or refractory disease and need urgent focused attention from pediatricians. Although MPP is currently recognized as a self-limiting illness, both groups of children in this study were at high risk of adverse long-term outcomes, including bronchiolitis obliterans, plastic bronchitis, and impaired lung function [15,16]. Bronchoscopy is a critical diagnostic modality for the management of respiratory diseases in children. With advances in bronchoscopic techniques for pediatric pulmonary disorders, interventional therapy via BAL has shortened the treatment duration for children with MPP and reduced the incidence of adverse outcomes [17]. Current research suggests that the main pathogenic mechanisms of *M. pneumoniae* are direct inflammation-related injury and an excessive immune response [18,19]. BAL exerts a therapeutic effect primarily by removing mucus plugs directly and local inflammatory factors under bronchoscopy as well as by allowing local administration of drugs [20]. In clinical practice, for various reasons, some of these children require multiple BAL procedures. However, while providing treatment, BAL also increases the physical and psychological burden on children. This study investigated the clinical characteristics of children who underwent multiple BAL treatments and identified several risk factors for repeated BAL using multivariate regression analysis.

Formation of mucus plugs is a strong risk factor for multiple BAL procedures in children with MPP. Endobronchial mucus plugs are endogenous foreign bodies, and their development is closely associated with inflammation of the bronchial mucosa, abnormal mucus secretion, impaired mucociliary clearance, and excessive immune activation [21]. Studies have demonstrated that mucus plugs are associated with persistent fever in children with MPP. Persistent fever often indicates dysregulation of inflammatory cytokines, and accumulation of these mediators is a key factor driving mucus plug formation [22]. In this study, the duration of fever was significantly longer in children in the MTG than in those in the STG (10.17 ± 4.42 days vs. 6.16 ± 3.73 days). Mucus plug formation can also directly induce atelectasis and regional impairment of ventilation, which may progress to respiratory failure in severe cases [23]. Both the process of mucus plug formation and its outcomes exacerbate the clinical symptoms of MPP in children, making the condition more complex. Simple drug therapy is not only slow-acting but also ineffective in preventing disease progression; failure to promptly remove this “foreign body” material can easily lead to more severe complications, such as necrotizing pneumonia and pleural effusion [24]. At this point, BAL becomes crucial. The main reason for performing multiple BAL treatments is that a single bronchoscopic procedure is insufficient to completely remove mucus plugs or that new mucus plugs form and block the tracheal lumen after removal. In the present study, the proportion of children with mucus plug formation was markedly higher in the MTG than in the STG (82.7% vs. 18.7%). Furthermore, the proportions of children with complications such as pulmonary consolidation (68.2% vs. 36.5%), pleural effusion (51.9% vs. 8.9%), and atelectasis (34.6% vs. 4.1%) were also significantly higher in the MTG than in the STG. Multivariate analysis revealed that mucus plug formation independently increased the risk of children requiring multiple BAL treatments by 13-fold (odds ratio 13.756).

Elevated CRP, LDH, and D-dimer levels are further risk factors for multiple BAL treatments in children with MPP. CRP, LDH, and D-dimer are important markers of tissue damage in children. They activate the coagulation cascade and promote a hypercoagulable state, which may contribute to the development of complications, such as the formation of bronchial mucus plugs and plastic bronchitis [25]. Li et al.[26] demonstrated that the degree of D-dimer elevation is positively correlated with the severity of MPP and that a serum D-dimer level of > 3.705 mg/L can serve as an independent predictor of MPP complicated by necrotizing pneumonia in children. Elevated levels of the above three indicators reflect a more severe inflammatory state and tissue damage in children. Such children often develop more refractory disease during treatment, and multiple BAL interventions can better control disease progression by lavaging locally inflammatory factors and delivering targeted medications via bronchoscopy.

This study has several limitations. First, it only included pediatric patients from Fuyang People's Hospital. As a single-center study, its findings have limited generalizability. Second, patients who were unwilling to undergo bronchoscopy and those with incomplete data were excluded. Therefore, the possibility of selection bias cannot be excluded. Third, there is no consistent objective indicator that can be used to evaluate the condition of pediatric patients when recommending a second BAL procedure. Although these children are assessed at the same time by two pediatricians with an attending physician or higher qualification to reduce the risk of subjective judgment based on symptoms, such bias cannot be ignored. Further studies with larger sample sizes are required to confirm these findings.

In summary, mucus plug formation and elevated CRP, LDH, and D-dimer levels are strong risk factors for multiple BAL procedures in children with MPP. Early identification of such children is necessary in clinical practice. Anti-inflammatory medication should be administered promptly to halt the progression of inflammatory damage, thereby minimizing the distress caused by repeated BAL. Regular monitoring for changes in the aforementioned indicators is also necessary, and repeat BAL should be considered only when medication is insufficiently effective.

## Data availability statement

The datasets used and analyzed during the current study are available from the corresponding author on reasonable request.

## Funding information

This work was supported by the Horizontal Medical Research Special Cultivation Project of Fuyang Normal University (2024FYNUEY08).

## Ethics approval and consent to participate

The study protocol was designed in accordance with the Declaration of Helsinki and approved by the Ethics Committee of Fuyang People's Hospital (Ethics number: 2025-108). Given the retrospective nature of the study, the requirement for informed consent was waived by the same ethics committee.

## Conflicts of interest

The authors declare no conflict of interest.
